# Effect of Acupuncture on Muscle Endurance in the Female Shoulder Joint: A Pilot Study

**DOI:** 10.1155/2020/9786367

**Published:** 2020-09-04

**Authors:** I.-Lin Wang, Yi-Ming Chen, Rui Hu, Jun Wang, Zheng-Bin Li

**Affiliations:** ^1^College of Physical Education, Hubei Normal University of Huangshi, Hubei 435002, Huangshi, China; ^2^Graduate Institute, Jilin Sport University of Changchun, Jilin 130022, China; ^3^School of Human Movement Science, Jilin Sport University of Changchun, Jilin 130022, China

## Abstract

Shoulder joint dysfunction is the leading cause of decreased athletic ability in athletes. Shoulder joint sports injuries affect the athletic performance of athletes. Improvements in the muscle endurance of the shoulder joint can reduce the incidence of shoulder joint dysfunction. Acupuncture has been an important part of Asian culture for a long time. In acupuncture, nerves are stimulated, inducing postactivation potentiation (PAP) in the body's motor units and enhancing muscle strength. In this research, 20 female participants with full flexion/extension and adduction/abduction ranges of motion in the shoulder joint during isokinetic exercises underwent stimulation of the following acupuncture points in the shoulder joint: Binao (LI14), Jianyu (LI15), Jianliao (SJ14), Naohui (SJ13), Yuzhong (KI26), Zhongfu (LU1), Yunmen (LU2), Xiabai (LU4), Chize (LU5), Tianfu (LU3), and Xiaoluo (SJ12). In the study, there were significant increases after acupuncture in the average maximum torque in flexion, extension, and adduction; the average work in flexion/extension and adduction/abduction; the average power in flexion/extension and adduction/abduction; the total work in flexion/extension and adduction/abduction; the total net sagittal-plane work (flexion + extension); and the total net frontal-plane work (adduction + abduction) (*P* < 0.05). The average maximum abduction torque did not increase significantly, potentially due to antagonistic forces of muscles. Therefore, acupuncture at acupoints around the shoulder joint can increase muscle excitability, thereby delaying muscle fatigue and increasing muscle endurance.

## 1. Introduction

Shoulder osteoarthritis may be caused by shoulder muscle fatigue and frequent upper limb movements. Previous studies on shoulder injuries have indicated that 91.3% of young swimmers suffer from shoulder pain [1]. The shoulder injury rate in rugby players is 55.6% [2], and shoulder injuries in senior high school baseball players account for 17.6% of all reported injuries [3]. Shoulder pain can be caused by prolonged exercise and flexibility or muscle imbalances between internal and external rotators during upper limb movements [2]. In addition, repeating or continuing maximum voluntary contractions for a long time increases motor unit recruitment and fatigue [4, 5], which result in instability and changes in the muscles around the scapulae, glenohumeral joint, and shoulder-thorax joint [6]. Shoulder joint dysfunction causes athletes to discontinue athletic participation and end a season or career early [7]. In addition, frequent upper limb movements lead to functional impairment of the shoulder in athletes with glenohumeral joint laxity, biceps tendinitis, or rotator cuff injury and can lead to shoulder joint impingement syndrome [8]. Previous studies have shown that people who participate in endurance training have a high level of muscle fatigue resistance [9]. Enhancing muscle endurance can improve shoulder joint stability and prevent shoulder fatigue. In addition, endurance training can lead human skeletal muscles to adapt to sports activities performed repeatedly over long periods of time and improve the exercise capacity of relevant limbs [10]. Therefore, frequent upper limb movements may cause muscle fatigue around the shoulder joint, improve the muscle endurance of the shoulder joint, reduce shoulder pain or the risk of other shoulder joint diseases caused by such fatigue, and prevent injury to the shoulder joint causing functional impairment.

PAP can stimulate the body, leading to enhanced nerve excitability, increased motor unit recruitment, increased central nerve inhibition, and reduced resulting muscle strength [11]. PAP allows the body to activate motor neurons through high-intensity sports and leads to synapse excitement in the spinal cord [12]. PAP enhancement causes nervous system excitement and enhanced contractile function [13], and the central nervous system reflexively increases the strength of muscles in the body. Past studies have shown that rugby players' upper limb strength increases after performing bench press training [14]. The same upper limb strength-induced PAP phenomenon has been observed in martial arts athletes after performing bench press exercises [15]. The PAP phenomenon was also observed in endurance athletes after maximal voluntary contractions due to increased muscle recruitment [16]. The study showed that fatigue resolution is associated with increased muscle strength and optimal athletic performance, which are attributed to the PAP effect being caused by increased excitability of *α*-motor neurons, as reflected by H-reflex changes in neurons [17]. Therefore, the body's nerves are stimulated, and the excitability of neurons increases, which may lead to PAP. At this time, the body will show increased strength if fatigue regression does not occur, and long periods of exercise may be conducive to increasing physical endurance.

In addition, acupuncture can alleviate physical pain and relieve symptoms of chronic diseases, and in athletes, it can relieve muscle fatigue, strengthen muscles, and quickly restore morphological functions [18]. Stimulating specific areas of the body can cause changes in excitement in the motor system and improve athletes' muscle performance [19]. For example, gait analysis studies in elderly patients undergoing acupuncture at specific points have shown that acupuncture at the Liangqiu point (ST34) can improve the efficiency of balance control and gait performance of elderly people [20]. Acupuncture at Hegu (LI4) and Shousanli (LI10) can enhance wrist extensor muscle activity [21] and increase the strength of the wrist extensors, causing motor nerve excitement. Acupuncture at Quchi (LI11) and Tianquan (PC2) can provide enough neuromuscular stimulation for the biceps brachii to promote the recruitment of motor units and improve the athletic ability [22]. Furthermore, acupuncture can trigger somatic autonomic reflexes and cause body vasodilation, thereby optimizing athletes' cardiovascular capacity and performance and improving athletic performance and recovery after the exercise [23]. Acupuncture at Neiguan (PC6) and Zusanli (ST36) can reduce muscle fatigue in basketball players and accelerate the recovery of their athletic abilities [23]. Therefore, acupuncture at specific areas of the body may improve the efficiency of balance control, relieve muscle fatigue, improve athletic performance, and accelerate the recovery of individuals' athletic abilities. To explore the effect of acupuncture on muscle endurance in the upper limb shoulder joint, an isokinetic instrument and EMG device were used for testing. We hypothesized that acupuncture can trigger a physiological response in the body that improves shoulder muscle endurance after isokinetic exercises of the shoulder joint.

## 2. Methods

### 2.1. Participants

Twenty healthy volunteer females from Jilin Sport University (age: 20.64 ± 0.69 years; body mass: 58.27 ± 11.03 kg; and height: 163.17 ± 5.07 cm) were recruited. All participants signed informed consent forms before they participated in the study. The exclusion criteria were as follows: a history of shoulder disorders or surgery, pregnancy, upper limb pain, neuromuscular impairments, and any aversion to needles, a history of acupuncture treatment within the last 4 weeks, and a history of using any medications. The participants were instructed to maintain their normal level of physical activity during the entire study period and to refrain from performing any form of physical exercise for at least 48 h prior to the tests. Food intake was restricted to a light meal 3 h before the tests, and beverages containing caffeine or alcohol were not allowed to be consumed within 12 h prior to the measurements [24, 25].

### 2.2. Instruments and Equipment

In this study, continuous data on flexion/extension and adduction/abduction forces of the shoulder joint were collected by an isokinetic Con-Trex dynamometer (Con-Trex MJ, Germany, Schnaittach, Physiomed). Disposable circular electrodes with a 10 mm diameter and a six-channel, portable electromyography device (BTS FreeEMG 300, BTS S.p.A., Milan, Italy) were used to collect electromyographic signals before and after acupuncture. The sampling frequency was 1000 Hz. Disposable sterile needles (0.25 mm *∗* 40 mm, Suzhou Medical Appliance Factory, Suzhou, People's Republic of China) were used for acupuncture.

### 2.3. Procedures

In brief, we introduced each participant to the procedure, described the effort required, and provided the same instructions that were used to begin and end each testing sequence to familiarize them with the experimental setup, equipment, and procedures. Then, the participants sat on an isokinetic dynamometer, and their lower body, waist, and thighs were stabilized using specific straps to control for extraneous body movements. The angle between the seatback and seat was adjusted to 85°, the rotation angle was adjusted to 15°, the shoulder was extended at 90°, the forearm was in a neutral position, the rotation axis of the isometric head was aligned with the center of the shoulder joint, and the shoulder joint was actively rotated from 0° to 60° to the maximum range of motion (ROM) during the motion tasks. Each participant was required to complete shoulder isokinetic muscle strength tests, including 1 pretest and 1 posttest.

The participants were randomly selected. In the preliminary experiment, 5 repetitions of flexion/extension and abduction/adduction tests were performed at a speed of 30°/s to familiarize the participants with the experimental process. Afterwards, the subjects could rest for three minutes. During the break, the participants' skin was cleaned with 75% alcohol and shaved, and the skin could dry for 2 minutes. The electrodes were then placed near the pectoralis major (PS), anterior deltoid (DA), posterior deltoid (DP), infraspinatus (ID), and triceps brachii (TC) shoulder muscles for muscle activity recording. The experiment began, and the participants performed the same, the shoulder joint flexion/extension and adduction/abduction, tasks 5 times at a speed of 60°/s for the pretest. Afterwards, the participants rested for 3 minutes. Then, experienced acupuncturists used disposable sterile needles to stimulate the acupoints around the shoulder joint.

### 2.4. Acupuncture

**Figure 1:**
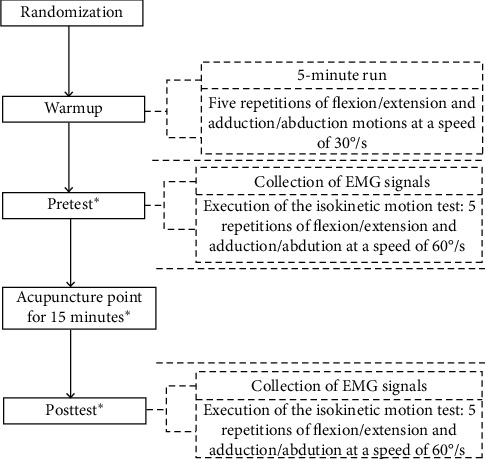
Experimental protocol.

The acupuncture points used in the study were Binao (LI14), Jianyu (LI15), Jianliao (SJ14), Naohui (SJ13), Yuzhong (KI26), Zhongfu (LU1), Yunmen (LU2), Xiabai (LU4), Chize (LU5), Tianfu (LU3), and Xiaoluo (SJ12). These points were chosen because they act as proximal points for joint movement, and acupuncture at these points can activate proximal muscles [26]. Acupuncture was performed at each acupoint for 15 minutes, and twisting, complementary, and reduction techniques were performed at 2 minutes, 5 minutes, and 10 minutes after acupuncture. Afterwards, the participants completed 5 repetitions of shoulder flexion/extension and adduction/abduction isokinetic motions at 60°/s for the posttest. The experimental process is shown in Figure 1.

### 2.5. Surface Electromyographic (sEMG) Recording

The electromyographic activity was recorded by the FREEEMG 300 EMG device (BTS, Italy), which is a portable unit for collecting and transmitting electromyographic data wirelessly to the computer. A sampling frequency of 1000 Hz was used. Preamplifiers located next to the measuring electrodes allowed us to rule out the influence of wire movement on the measurements. The muscles that surround the shoulder joint and help it move in all directions were selected. The program was executed at the same time as the isokinetic movement, and the sEMG signals were synchronized in the data collection.

### 2.6. Data Analysis

The Con-Trex isokinetic dynamometer was used to output the average maximum torque in flexion/extension and adduction/abduction, the average work in flexion/extension and adduction/abduction, the average power in flexion/extension and adduction/abduction, the total work in flexion/extension and adduction/abduction, the total net sagittal-plane work (flexion + extension), and the total net frontal-plane work (adduction + abduction) of the shoulder joint. For the analysis of the sEMG signals, the first second of each step (considered the transition time between force levels) was discarded, and the subsequent 5 s was used to determine the maximum voluntary contraction (MVC). All EMG signals were processed by performing specific routines carried out in MATLAB (version 7.0, MathWorks Inc., Natick, Massachusetts, United States). The EMG signals are reported as MVC%.

### 2.7. Statistical Analysis

SPSS20.0 software (Inc., Chicago, IL, USA) was used to analyze the isokinetic data of the shoulder joint in flexion/extension and adduction/abduction before and after acupuncture. The data are presented as the mean and standard deviation (SD). The paired *t*-test was used to compare the flexion/extension and adduction/abduction test values for the shoulder joint before and after acupuncture. *P* < 0.05 was set as the level of significance.

## 3. Results

### 3.1. Kinematic Index of Shoulder Joint Flexion and Extension

An isokinetic instrument was used to analyze the changes in parameters during flexion and extension of the shoulder joint. Therefore, we analyzed different parameters according to the motion state (Table 1). In the posttest assessment, the average maximum flexion torque normalized to body weight (kg) and the average maximum extension torque normalized to body weight (kg) significantly increased following acupuncture (+Δ56% and +Δ75%, *P* < 0.001). The average normalized work in flexion and the average normalized work in extension significantly increased following acupuncture (+Δ69% and +Δ38%, *P* < 0.001). The average power in flexion and the average power in extension significantly increased following acupuncture (+Δ70% and +Δ39%, *P* < 0.001). The total work in flexion and the total work in extension significantly increased following acupuncture (+Δ70% and +Δ39%, *P* < 0.001). The total work (flexion + extension) increased following acupuncture (+Δ56%, *P* < 0.001).

### 3.2. Kinematic Index of Shoulder Joint Adduction and Abduction

Table 2 shows the parameters assessed for adduction and abduction movements of the shoulder joint. The parameters before and after acupuncture are compared. The average maximum normalized adduction torque significantly increased following acupuncture (+Δ82%, *P* < 0.001). The average normalized adduction work and the average normalized abduction work significantly increased following acupuncture (+Δ98% and +Δ58%, *P* < 0.001). The average normalized adduction and abduction powers significantly increased following acupuncture (+Δ95% and +Δ58%, *P* < 0.001). The total adduction and abduction work significantly increased following acupuncture (+Δ95% and +Δ51%, *P* < 0.001). The total work (abduction + adduction) increased following acupuncture (+Δ74%, *P* < 0.001). There was no significant difference in the average maximum normalized abduction torque in the shoulder joint.

### 3.3. sEMG Signals for Shoulder Joint Flexion/Extension and Adduction/Abduction

**Figure 2:**
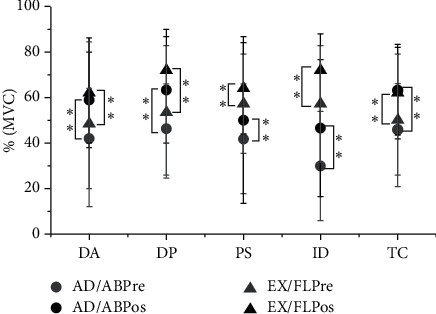
EMG data collected before and after acupuncture during flexion/extension and adduction/abduction of the shoulder joint. Note: 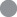: muscle activity of the shoulder joint during the adduction and abduction (AD/AB) exercise before acupuncture; 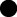: muscle activity during shoulder adduction and abduction (AD/AB) after acupuncture; 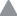: muscle activity of the shoulder joint during extension and flexion (EX/FL) before acupuncture; 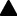: muscle activity during extension and flexion (EX/FL) after shoulder joint acupuncture. Data are expressed as the mean ± SD with five muscles in each group. The significance level (^*∗∗*^) was set to be *P* < 0.05. DA = deltoid anterior; DP = deltoid posterior; PS = pectoralis major; ID = infraspinatus; TC = triceps brachii.

EMG electrodes were placed on muscles around the shoulder joint throughout the isokinetic motion test to record the activity of these muscles. The results showed that the activity of the flexion/extension and adduction/abduction muscles increased significantly after acupuncture. There were significant differences between the flexor and extensor muscles of the shoulder joint following acupuncture: DA (+Δ28%, *P* < 0.01), DP (+Δ25%, *P* < 0.01), PS (+Δ23%, *P* < 0.01), ID (+Δ35%, *P* < 0.01), and TC (+Δ25%, *P* < 0.01). There were also significant differences between shoulder abductors and adductors: DA (+Δ37%, *P* < 0.01), DP (+Δ38%, *P* < 0.01), PS (+Δ26%, *P* < 0.01), ID (+Δ33%, *P* < 0.01), and TC (+Δ34%, *P* < 0.01).  Figure 2 shows the EMG data for flexion/extension and adduction/abduction of the shoulder joint before and after acupuncture.

## 4. Discussion

### 4.1. Analysis of the Kinetic Data before and after Acupuncture

After acupuncture, the average maximum flexion, extension, and adduction torque of the shoulder joint generated by the muscles increased. Previous studies have shown that acupuncture may cause the activation of peripheral muscle receptors and increase the number of activation receptors, leading to an increase in central nervous system excitability and the physiological response of “Deqi” in the muscles and peripheral motor neurons at the acupuncture site [27]. The complex sensory pattern in the “Deqi” response suggested that acupuncture may cause extensive stimulation of myelinated and unmyelinated nerve fibers [28]. Acupuncture stimulates nerves, thereby increasing the recruitment of motor units and inducing the nervous system to increase muscle activity; thus, muscle strength increases, and physical performance improves [29]. Therefore, the average maximum torque of the shoulder joint during the isokinetic motion increased after acupuncture, which may have been caused by the stimulation of nerves by acupuncture causing the physiological response of “Deqi.” However, the average maximum torque of the abductor muscles decreased after acupuncture, possibly because the active muscles and the antagonist muscles performed the same action. The central nervous system controls antagonist muscles to produce movements that compensate for movement impairments caused by insufficient muscle strength [30]. Previous studies have shown that, during continuous submaximal isometric contractions, the triceps brachii is the antagonistic muscle group, and the biceps, brachioradialis, and brachii are the active muscle group, and as the muscle activity in these muscle groups increases, the forces are counterbalanced [31]. Therefore, the decrease in the average maximum torque of the abductor muscles may have been caused by insufficient strength of the active abductor muscles, including the PS and TC, due to acupuncture being performed at the KI26, SJ12, LU5, LU2, and LU1 points; acupuncture at the LI14, LI15, SJ13, LU4, SJ14, and LU3 points caused the antagonist muscle group, including the DA, DP, and ID abductor muscles, to increase in strength, producing antagonistic muscle forces and weak extension forces, so the average maximum torque did not increase.

The results of this study showed that the average work, the average power, the total work in flexion/extension and adduction/abduction, the total net sagittal-plane work (flexion + extension), and the total net frontal-plane work (adduction + abduction) of the shoulder joint significantly increased after acupuncture. Past studies have shown that acupuncture stimulation of the somatosensory system can increase excitability of the spinal cord and superior spinal nerve muscles and that the recruitment of motor units increases muscle strength [24]. Induced stimulation of nerves may increase the strength of muscles in the body and induce the PAP phenomenon. The PAP phenomenon may increase the capacity of myosin light-chain kinase, which causes the phosphorylation of the myosin light chain to increase the Ca^2+^ sensitivity in the body, resulting in enhanced muscle strength [17, 32]. Therefore, the results of this study suggest that acupuncture may induce a neurophysiological response in the body, enhance the body's strength, and increase the average work, average power, total work in flexion/extension and adduction/abduction, total net sagittal-plane work (flexion + extension), and total net frontal-plane work (adduction + abduction) of the shoulder joint. In addition, past studies have shown that acupuncture at Xiaohai (SI8) and Jianwaishu (SI14) can prolong trapezius muscle activation and increase the strength of bilateral trapezius muscles in the body [25]. Therefore, acupuncture on the body may prolong the activation of muscles around the points and increase muscle strength. In this study, acupuncture at KI26, LU2, and LU1 may have enhanced the strength of the PS abductor muscle. Acupuncture at SJ12 and LU5 may enhance the abductor muscle group including the TC. Acupuncture at LI14 may enhance the DA adduction muscle. Acupuncture at LI15, SJ13, and SJ14 may enhance the DP adduction muscle. Acupuncture at LU4 and LU3 may strengthen the ID adduction muscle. Acupuncture may strengthen the shoulder joint abductor muscle group, including the PS and TC, and the adduction muscle group, including the DA, DP, and ID muscles. Therefore, the average work in adduction/abduction, the average power in adduction/abduction, the total work in adduction/abduction, and the total net frontal-plane work (adduction + abduction) increased. In addition, this study showed that acupuncture at LI14 may increase the strength of the DA flexor muscle. Acupuncture at KI26, LU2, and LU1 may increase the strength of the PS flexor muscle. Acupuncture at LI15, SJ13, and SJ14 may increase the flexibility of the DP muscle. Acupuncture at LU3 may increase the flexibility of the TC muscle. Thus, the flexion muscles, including the DA, PS, and extension muscles, including the DP and TC, were enhanced after acupuncture, potentially leading to the increase in the average work in flexion/extension, the average power in flexion/extension, the total work in flexion/extension, and the total net sagittal-plane work (flexion + extension) of the shoulder joint.

### 4.2. Analysis of Surface EMG Signals before and after Acupuncture

The results showed that the EMG signals of the flexor/extensor and abductor/adductor muscles increased after acupuncture. Past studies have shown that acupuncture may stimulate sensory receptors on the surface of the body, inducing muscle electromyography active substances and increasing through the afferent center, resulting in increased recruitment of motor units or the effective activation of muscles by electrical discharges to promote the activation of muscles [33]. Effective acupuncture can increase muscle activation during exercise to produce greater muscle strength and increase EMG signal amplitudes. Past studies have shown that the stimulation of specific acupoints around the muscles of the body may cause sensory and motor responses in the body, resulting in increased muscle activity and enhanced EMG signals to generate bilateral communication between neurons in the spinal cord, sensory neurons, and the upper spinal cord center [34]. It was shown that manual acupuncture performed at Shangjuxu (ST36) and Xiajuxu (ST39) of the tibial anterior muscle can activate body surface receptors and lead to enhanced neuronal activity in the body, leading to significantly increased ankle dorsiflexion muscle strength and contralateral body muscle strength and increased EMG signals [35]. Acupuncture at Zusanli (ST36) in the lower leg can improve the excitability of sensory nerves and increase muscle activity in athletes, which strengthens flexor and extensor muscles and increases EMG signals [36]. Transcutaneous electrical nerve stimulation (TENS) can be used as acupuncture on the calf points, Zusanli (ST36), Chengshan (BL57), Yanglingquan (GB34), and Sanyinjiao (SP6), to stimulate the sensory nerve fiber response and increase quadriceps muscle activity, thereby strengthening the muscles and increasing the EMG signals [19]. Therefore, acupuncture on muscles around points may cause neurogenic reactions leading to increased muscle activity, increased muscle strength, and increased EMG signals. In this study, after acupuncture was performed on the PS, DA, DP, ID, and TC muscles, it was observed that stimulation of a muscle group may cause reflexive activation of the muscles involved in the nerve response, increasing the EMG signal of the muscles at the stimulated site and the strength of the shoulder muscles used during adduction/abduction and flexion/extension exercises.

Acupuncture at KI26, LU2, and LU1 may activate the PS abductor muscle. Acupuncture at LU3 and LU5 activates the TC abductor muscle. Acupuncture at LI14 may activate the DA adductor muscle. Acupuncture at LI15, SJ13, and SJ14 may activate the DP adductor muscle. Acupuncture at LU4 and LU3 may activate the ID. After acupuncture, the abductor muscles of the shoulder joint, including the PS and the TC, as well as the adductor muscles, including the DA, DP, and ID, are activated, increasing the muscle strength and EMG signals. Moreover, this study showed that acupuncture at LI14 may cause DA extension muscle activation and that acupuncture at KI26, LU2, and LU1 activates the PS flexor muscle. Acupuncture at LI15, SJ13, and SJ14 may activate the DA flexor muscle; acupuncture at LU3 may activate the TC extensor muscle. After acupuncture, the flexion muscle group, including the DA and PS of the shoulder joint, and the extensor muscle group, including the DP and TC, are activated, and the flexion and extension exercise intensity is increased to increase the EMG signal. Therefore, the central nerve reflex induced by sensory nerve stimulation after acupuncture leads to a change in the potential caused by muscle contractions, which leads to increased muscle strength and EMG signals at the stimulated site.

Our research has certain limitations. First, we did not measure muscle blood flow to study the mechanism of acupuncture. Second, the subjects were not blinded to the type of intervention, and we did not study the effects of sham acupuncture. Therefore, a possible placebo effect cannot be ruled out. We expect to address these questions in our future research.

## 5. Conclusion

When used as an auxiliary training method, acupuncture can increase individuals' exercise capacity and obviously regulate the strength and endurance of human skeletal muscles. Acupuncture can delay declines in strength and endurance and prevent exercise fatigue. Acupuncture at the body's receptors to increase muscle activity may induce PAP and increase muscle strength. Acupuncture at points around the shoulder joint may cause muscle contractions, thereby increasing muscle strength and improving physical performance. Therefore, acupuncture may improve muscle endurance by strengthening muscles, delaying muscle fatigue, and extending the amount of time an individual can exercise. Additional studies need to be conducted to examine the effect of acupuncture on the time effectiveness of muscle endurance.

## Figures and Tables

**Table 1:** Comparison of the isokinetic flexion/extension results before acupuncture and after acupuncture.

Flexion/extension (sagittal plane)	Pre60		Post60		*P*
	Mean	SD	Mean	SD	
Average maximum torque flexion/kg (Nm/kg)	0.600	0.300	0.939	0.249	<0.001
Average maximum torque extension/kg (Nm/kg)	0.329	0.209	0.557	0.233	0.005
Average work flexion/kg (J/kg)	0.792	0.389	1.339	0.406	<0.001
Average work extension/kg (J/kg)	0.621	0.239	0.862	0.229	<0.001
Average power flexion/kg (W/kg)	0.384	0.194	0.653	0.197	<0.001
Average power extension/kg (W/kg)	0.303	0.123	0.422	0.113	<0.001
Total net work flexion (J)	292.731	120.382	498.955	110.074	<0.001
Total net work extension (J)	230.061	70.138	321.016	58.336	<0.001
Total work (flexion + extension) (J)	522.797	187.457	819.971	137.097	<0.001

*Note*. Differences were considered significant when *P* < 0.05.

**Table 2:** Comparison of the isokinetic adduction/abduction results before and after acupuncture.

Adduction/abduction (frontal plane)	Pre60		Post60		*P*
	Mean	SD	Mean	SD	
Average maximum torque adduction/kg (Nm/kg)	0.501	0.244	0.915	0.180	<0.001
Average maximum torque abduction/kg (Nm/kg)	0.422	0.166	0.414	0.235	0.895
Average work adduction/kg (J/kg)	0.666	0.330	1.321	0.277	<0.001
Average work abduction/kg (J/kg)	0.606	0.165	0.939	0.163	<0.001
Average power adduction/kg (W/kg)	0.303	0.164	0.643	0.144	<0.001
Average power abduction/kg (W/kg)	0.281	0.082	0.445	0.087	<0.001
Total work adduction (J)	232.317	111.254	454.532	97.486	<0.001
Total work abduction (J)	210.573	65.843	319.229	34.889	<0.001
Total work (adduction + abduction) (J)	442.891	167.626	773.762	119.997	<0.001

*Note*. Differences were considered significant when *P* < 0.05.

## Data Availability

The data used to support the findings of this study are included within the article.
